# Impact of drought on crime in California: A synthetic control approach

**DOI:** 10.1371/journal.pone.0185629

**Published:** 2017-10-04

**Authors:** Dana E. Goin, Kara E. Rudolph, Jennifer Ahern

**Affiliations:** Division of Epidemiology, School of Public Health, University of California, Berkeley, Berkeley, California, United States of America; University of Rijeka, CROATIA

## Abstract

Climate and weather have been linked to criminal activity. The connection between climatological conditions and crime is of growing importance as we seek to understand the societal implications of climate change. This study describes the mechanisms theorized to link annual variations in climate to crime in California and examines the effect of drought on statewide crime rates from 2011–2015. California has suffered severe drought since 2011, resulting in intensely dry winters and several of the hottest days on record. It is likely that the drought increased economic stress and shifted routine activities of the population, potentially increasing the likelihood of crime. We used a synthetic control method to estimate the impact of California’s drought on both property and violent crimes. We found a significant increase in property crimes during the drought, but no effect on violent crimes. This result was robust to several sensitivity analyses, including a negative control.

## Introduction

The effect of climate on conflict and criminal activity has been demonstrated across the world. Substantial work in recent years has linked major shifts in climate to increases in crime, violence, and political upheaval, particularly in places also experiencing economic instability or in which personal livelihoods depend on agricultural production[1–11]. Beyond major climatic shifts, research also suggests daily and seasonal variation in weather patterns can impact crime rates [6,12–14]. High temperatures, precipitation, wind speed, and humidity have all been tied to changes in crime patterns [2,6,13,15–24]. The links between climatological conditions and crime are of growing importance as the scientific community seeks to understand the full societal implications of climate change. Understanding these relationships is particularly important in places like California, where climate change is anticipated to have major impacts on the agricultural economy and quality of both urban and rural life.

California suffered from a severe drought from 2011 to 2015, prompting Governor Jerry Brown to proclaim a drought emergency in 2014 and enact historic statewide water restrictions in 2015 [25,26]. The drought resulted in several of the driest winters on record across the state, and accompanied heat waves that yielded record temperatures [27]. To our knowledge, no studies to date have attempted to identify the impact of this historic drought on crime. To fill this research gap, this paper will discuss mechanisms that link drought to crime and quantify the impact of drought on property and violent crime rates in California. As California and the rest of the country look to a future that includes periods of drought [28], the impact on crime is important to understand so policymakers, police agencies, and communities can prepare.

## Theoretical framework

There are two main theoretical mechanisms linking drought to crime rates. First, major climatic shifts have economic and social consequences, which can subsequently influence crime. Second, alterations in daily weather patterns can impact human behavior and routine activities, modifying risk of victimization. While our focus is on the overall impact of drought on crime in California, some consideration of the micro-level mechanisms that may underlie these effects supports the plausibility of the hypothesis, and explicates some of the potential pathways encompassed in this overall effect.

### Drought and economic stress

The drought, which began in 2011, was a major climactic event for California. While several other states also suffered from drought during this time, California’s experience was the most severe and persistent [29,30]. The scale of the agricultural industry in California increases the political and economic stakes of drought, as its aquaculture, farming, ranching, and dairy industries use the majority of surface and groundwater resources within the state [31]. During the peak of the drought in 2014, 75% of California’s range and pastureland was rated in poor condition, and many farmers fallowed portions of their land, especially in the Central Valley [32–34]. However, the drought did not devastate the agricultural economy as much as predicted, mainly due to the overdrawing of groundwater sources [32,35]. This mitigated some of the short-term economic losses that drought might have caused, but is unsustainable [36].

The relatively modest overall economic impact of the drought does not mean there were no effects on local communities. Indeed, Central Valley farmers pulled so much groundwater that predominantly low-income communities in neighboring areas found their wells drying up [37–39]. Water rates rose for urban and semi-urban consumers, even as they sought to conserve [40]. The increase in water prices and drought surcharges hit lower-income families hardest. Families with annual earnings less than $25,000 per year saw their water prices increase to over 2% of household income, exceeding affordability thresholds set by the State of California and the Environmental Protection Agency [41]. The disappearance of well water in rural communities and higher water prices in urbanized areas illustrate how the drought disproportionately affected the less wealthy across the state [42,43].

Increases in unemployment, greater inequality, declining wages, and other forms of political or economic disempowerment can erode community cohesion and foment divisiveness, resulting in higher crime [44]. Additionally, many of these factors shift perceptions about fairness, equality, and support, resulting in altered patterns of individual behavior and motivations regarding crime. Drought-related economic stress may have exacerbated these issues faced by many Californians, especially those living in areas highly dependent on groundwater and those burdened by drought surcharges and regulations.

### Drought and routine activities

Individual motivation is a key determinant of criminal action and victimization, but it is not the only factor that influences crime incidence. Crimes require a confluence of events in order to occur: there must be a motivated actor, an opportunity to identify and exploit an available target, and a lack of witnesses or guardianship. While individual motivation plays an important role, this routine activity theory of crime also suggests that changes in the daily activities of other people can alter the window of opportunity for criminal action [45]. This theory is often employed to describe how weather affects rates of both property and violent crime. The temperature, humidity, precipitation, and the strength of winds on any given day can all impact the likelihood of people spending time outdoors, travelling, or going to social activity spaces during the day or at night, and such activities have implications for the potential for both property and violent crime [14].

We considered the effects of drought to encompass the economic consequences of drought, behavioral impacts of weather, and alterations of routine activities. These effects could be examined at several relevant temporal and geographic scales. This paper focuses on the drought as a climactic event that began in 2011 and persisted through 2015, and affected the entire state of California. Given the plausible micro-level mechanisms that would link drought with crime, we hypothesized that the California drought would be associated with increased rates of both property and violent crime from 2011–2015, as compared to the ten prior years.

## Methods

### Overview

The drought’s impact on determinants of crime likely included both daily changes in routine activities, monthly increases in residential water prices, seasonal job losses, and long-term restructuring of water rights and agricultural investment. In order to capture overall changes in patterns of criminality affected by each of these mechanisms, we used the annual state-level Uniform Crime Statistics maintained by the FBI. We chose to estimate the effects at the state-year level because while areas of California experienced the drought differently, the entire state felt at least some effects of drought starting in 2011, and therefore we needed other states to serve as controls. In addition, we chose to estimate the effects at the state level because sub-state crime estimates are not as reliable in terms of consistent reporting, as has been documented in previous validation studies [46–48]. We chose to examine yearly effects because this allowed us to focus on trends of crime without the additional variability from seasonal cycles that would be present in more fine-grained temporal data. Additionally, the state-level covariate data are only available at annual intervals, and the annual crime data are less affected from reporting inconsistencies.

We used a synthetic control method to estimate California’s expected rates of violent and property crime in the absence of drought, and compared these to the observed crime rates to quantify the drought’s impact on crime during 2011–2015 [49]. This approach has previously been used to estimate the effects of policy, social, and environmental changes [49–53]. Our hypothesis is that drought affects crime not as a climatic phenomenon alone, but as a change in climate that alters economic and social organization and behavioral patterns. To assess whether our results could be due to chance, we used a permutation test. We utilized a negative control to explore whether our results could be explained by co-occurring changes in California’s economy that were unrelated to the drought.

### Analysis

We use a synthetic control method developed by Abadie, Diamond, and Hainmueller in this analysis [52]. This method uses a weighted combination of states to create a synthetic “control” California, which provides an estimate of expected crime rates in the state if the drought had not occurred. The states that comprise California’s synthetic control are selected by the method based on their pre-2011 trends in covariate values and crime rates. Those that are best able to predict the pre-2011 crime trends in California are chosen for the synthetic control group. The expected crime rates for California from 2011–2015 in the absence of drought are then compared against the observed crime rates. A difference between the observed and expected values can therefore be interpreted as the impact of the drought on crime.

While traditional statistical inference techniques do not work with this approach, given the small sample size, a permutation test can be used to assess how unusual such an effect would be if it were due to chance and thus provide context for the effect size. This permutation test involves implementing the synthetic control technique for each state, as though it were the one that had experienced drought during 2011–2015. The estimated effect for California can then be compared to the size of these other effect estimates. Typically, the permutation test results are compared for states in which pre-drought crime trends are well predicted by the synthetic control. We will present results of the test for all states, in addition to the results for those states with 20, 5, and 2 times the mean squared prediction error (MSPE) observed for California. States with a poorly matched synthetic control might appear to have more extreme differences as an artifact of poor prediction.

It is possible that drought and unrelated economic changes occurred simultaneously in California. While we included a wide range of economic indicators as covariates in the synthetic control analysis, it is possible other economic factors we did not control for changed in 2011, and though unrelated to the drought, affected crime rates through 2015. Since the synthetic control analysis does not allow us to distinguish between co-occurring events, we used a negative control to investigate whether changes in the economy could be an alternative explanation for our results. A negative control estimates the effect of an exposure on an outcome that it should not plausibly impact, but which may be affected by a confounding factor [54]. If an effect is observed in such a situation, it can be assumed that there is confounding or bias in the study. We examined median cashier wages as a negative control. We will also present evidence from what we call a positive control, in which we estimate the effect of the exposure on an outcome that it should affect. Median farmworker wages served as the positive control. We expect the drought to have affected farmworker wages, since many farm owners faced financial pressures including rising water costs, and chose to offset this by suppressing wage increases or firing workers [32]. However, we would not expect the drought to impact cashier wages. An observed effect of drought on median cashier wages would indicate the co-occurrence of drought and unrelated economic changes in California, and would suggest that observed effects of drought on crime may be similarly contaminated. An observed effect of drought on farmworker wages, however, would support our theory that the drought had economic implications for the agricultural industry and would bolster our confidence that the method is able to detect drought-related changes. Observing an effect of the drought on farmworker wages, but not cashier wages, would provide additional evidence that drought’s effect on crime rates is not spurious.

### Data sources

We used state-level annual crime statistics from the FBI’s Uniform Crime Reporting program from 2000–2015, and divided these by estimates of the total population to create rates of crime for each state-year unit [55]. Property crime included burglary, larceny-theft, and motor vehicle theft. Violent crime comprised murder and non-negligent manslaughter, robbery, and aggravated assault. We excluded forcible rape because the definition changed in 2012 [48].

We used the following state-level covariates known to be predictive of crime or associated with economic characteristics in our analysis: distribution of age, race/ethnicity, and poverty; median income; median wages by industry; unemployment; population density; income inequality; jobs per capita; income per capita; average gas prices per gallon; law enforcement presence; housing affordability by income; migration; ratio of housing value to income level; and drug and alcohol use by age. Percentages of the population age 15–19, age 20–24, Asian, Black, or Hispanic were from the Census Bureau [56]. The percent of the population living under the poverty line, log of median income, the unemployment rate, the log of the population density, percent paying over 30% of their income on housing by income group, median wages by industry, percent staying in the same house, percent moving within a state, percent moving out of state, ratio of home value to income level for owner-occupied units, and the Gini index came from the American Community Survey [57]. Average gas prices per gallon were compiled from the U.S. Energy Information Administration [58]. The FBI’s Uniform Crime Reports provided the rate of law enforcement agencies and employees [55]. Income per capita and jobs per capita came from the United States’ Bureau of Economic Analysis [59]. Rates of illicit illegal drug use, binge alcohol drinking, and abuse or dependence of alcohol or illicit drugs by age group came from the National Survey on Drug Use and Health [60]. The negative control analysis used median cashier and farmworker wages from the Bureau of Labor Statistics Occupational Employment Statistics program [61].

Temperature changes often occur alongside drought and are also likely to impact crime. Thus, we performed a sensitivity analysis, which included average annual temperature as a control covariate. The data for temperature came from NOAA’s National Centers for Environmental Information [62].

## Results

**Fig 1:**
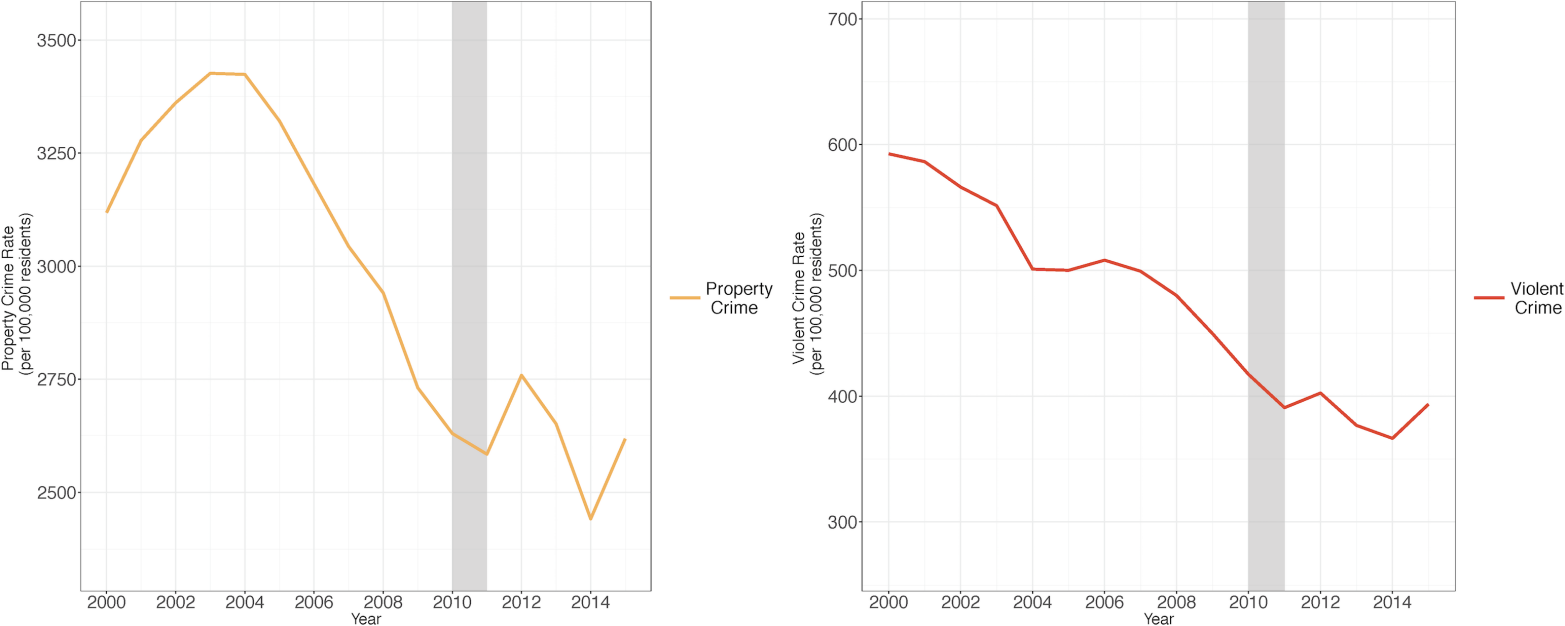
Property and violent crime rates by type in California, 2000–2015. Source: Property and violent crime rates are from the FBI Uniform Crime Reports from 2000–2015. Note: The grey bar indicates the beginning of the drought.

**Fig 2:**
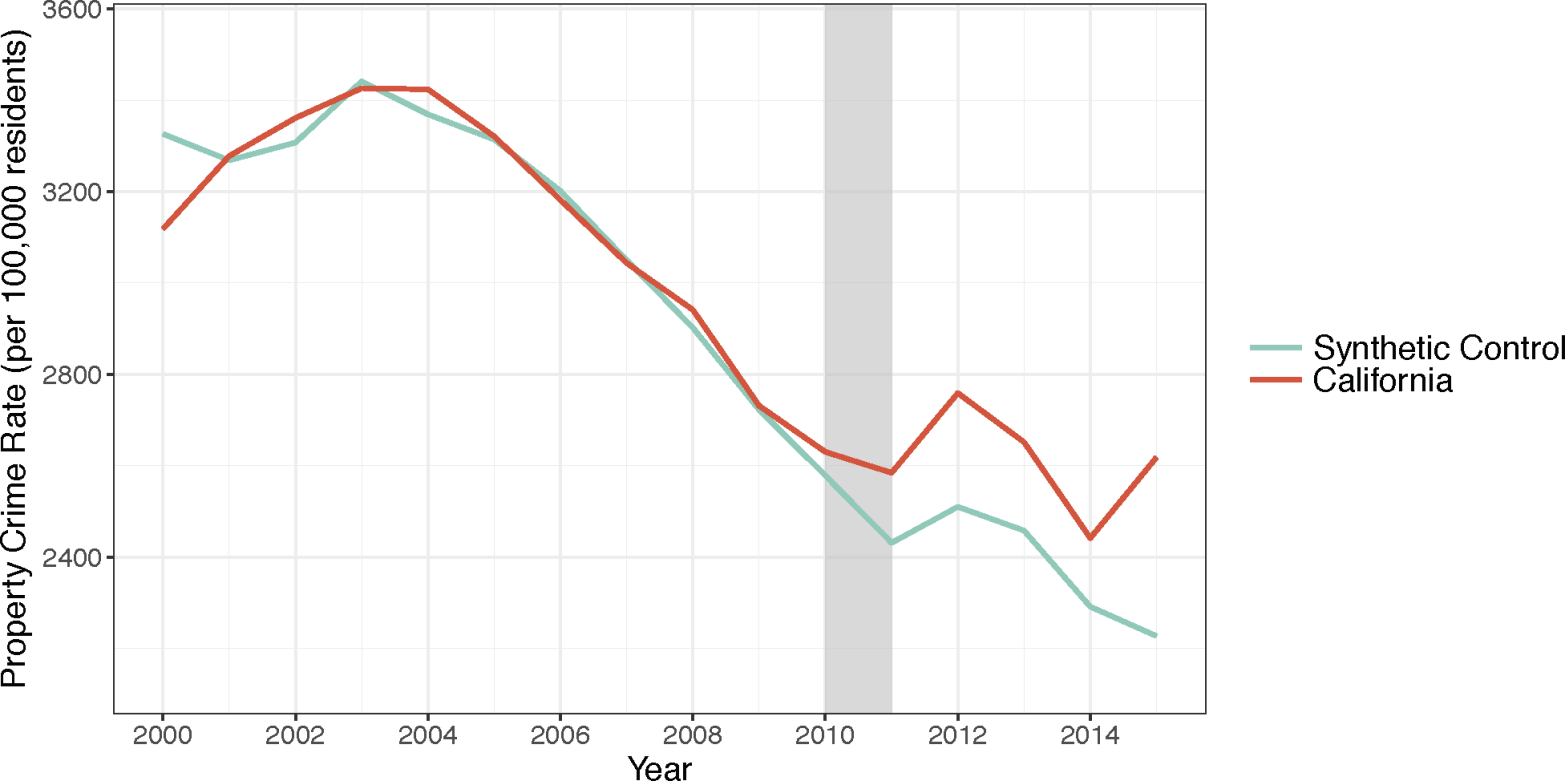
Property crime in California and its synthetic control. Source: Property crime rates are from the FBI Uniform Crime Reports from 2000–2015. Note: The grey bar indicates the beginning of the drought.

Property crime was much more common than violent crime in California, but both were in periods of decline from 2000–2010, the pre-drought period (Fig 1). The covariates used to calculate the weights for the synthetic control series are listed in Table 1, along with their values in California, the mean values for the rest of the states, and their values in both the property and violence synthetic controls. The states included in the synthetic controls are listed along with their weights in Table 2. The mean squared prediction errors (MSPE) in the pre-drought period for each type of crime are listed in Table 3. Overall the pre-drought property crime levels appear to be well approximated by the synthetic control (Fig 2).

**Table 1:** Comparison of mean covariate levels in California, its synthetic controls, and control states.

	Sample Mean	California	Synthetic Property Crime	Synthetic Violent Crime
Illicit Drug Use among 12–17 year olds (%)	9.91	10.43	10.40	9.79
Illicit Drug Use among 18–25 year olds (%)	20.74	21.31	21.62	21.15
Illicit Drug Use among people over age 26 (%)	6.08	7.19	6.66	6.11
Binge Alcohol Drinking Among People Over Age 12 (%)	23.50	21.82	24.16	23.20
Binge Alcohol Drinking Among 12–17 year olds (%)	9.62	8.98	9.72	9.66
Binge Alcohol Drinking among 18–25 year olds (%)	42.78	38.56	41.85	40.81
Binge Alcohol Drinking among people over age 26 (%)	21.95	20.48	23.04	21.89
People age 15–19 (%)	0.07	0.08	0.07	0.07
People age 20–24 (%)	0.07	0.07	0.07	0.07
People age 25–29 (%)	0.07	0.07	0.07	0.07
Asian or Pacific Islander, non-Hispanic (%)	0.02	0.12	0.06	0.05
Black or African American, non-Hispanic (%)	0.11	0.06	0.11	0.14
Hispanic (%)	0.09	0.35	0.22	0.21
White, non-Hispanic (%)	0.74	0.44	0.58	0.58
Log median income	10.91	11.05	10.94	10.89
Unemployment rate	5.53	6.96	5.99	5.79
Income per capita	34598.65	38738.09	38386.63	38417.93
Jobs per capita	0.59	0.56	0.58	0.56
Population density	172.18	218.35	177.38	272.16
People living in poverty (%)	13.20	13.63	13.59	14.50
Total number of law enforcement employees	19413.34	117829.0	54763.19	81190.08
Total number of law enforcement agencies	299.51	458.14	365.37	536.94
Gini index	0.45	0.47	0.47	0.49
Spend >30% of income on housing, who own home with income < $20,000/year (%)	0.05	0.04	0.04	0.05
Spend >30% of income on housing, who own home with income $35,000-$49,999 (%)	0.03	0.04	0.04	0.03
Spend >30% of income on housing, who own home with income $20,000-$34,999 (%)	0.04	0.04	0.04	0.04
Spend >30% of income on housing, who own home with income $50,000-$74,999 (%)	0.03	0.06	0.04	0.04
Spend >30% of income on housing, who own home with income >$75,000 (%)	0.02	0.08	0.04	0.04
Spend >30% of income on housing, who rent home with income <$20,000 (%)	0.08	0.09	0.10	0.10
Spend >30% of income on housing, who rent home with income $20,000-$34,999 (%)	0.04	0.07	0.06	0.06
Spend >30% of income on housing, who rent home with income $35,000-$49,999 (%)	0.01	0.04	0.03	0.02
Spend >30% of income on housing, who own home with income $50,000-$74,999 (%)	0.00	0.02	0.01	0.01
Spend >30% of income on housing, who own home with income >$75,000 (%)	0.00	0.01	0.00	0.00
Median earnings	38770.20	43415.17	40518.26	39891.43
Median earnings among those in managerial profession	53252.28	66984.50	57814.68	56710.33
Median earnings among those in service profession	24589.02	27870.83	27543.23	25771.75
Median earnings among those in sales profession	37519.29	41818.33	38955.61	39124.77
Median earnings among those in construction profession	38372.44	39219.83	41048.52	36947.16
Average price of gasoline per gallon	2.21	2.35	2.27	2.18
Percent living in the same house as the year before	0.84	0.84	0.83	0.86
Percent who moved from out of state in the past year	0.03	0.01	0.03	0.02
Percent who moved from abroad in the past year	0.01	0.01	0.01	0.01
Ratio of home value to income in owner occupied units < 2 (%)	32.71	8.10	27.96	30.79
Ratio of home value to income in owner occupied units 2 to 2.9 (%)	24.69	11.85	19.92	20.52
Ratio of home value to income in owner occupied units 3 to 3.9 (%)	15.08	14.17	14.42	13.77
Ratio of home value to income in owner occupied units > = 4 (%)	27.13	65.40	37.23	34.46
Average property crime rate from 2000–2010	3326.86	3132.35	3134.56	3196.85
Average violent crime rate from 2000–2010	380.45	513.84	513.15	514.27

**Table 2:** Weights assigned to states for California’s property and violent crime synthetic controls.

Property Crime		Violent Crime	
State name	Weight	State name	Weight
New York	0.395	Florida	0.289
North Carolina	0.44	North Carolina	0.503
Ohio	0.165	Ohio	0.207

**Table 3:** Mean squared prediction error in the pre-drought period (rate per 100,000).

Property Crime	4939.56
Violent Crime	70.25

**Fig 3:**
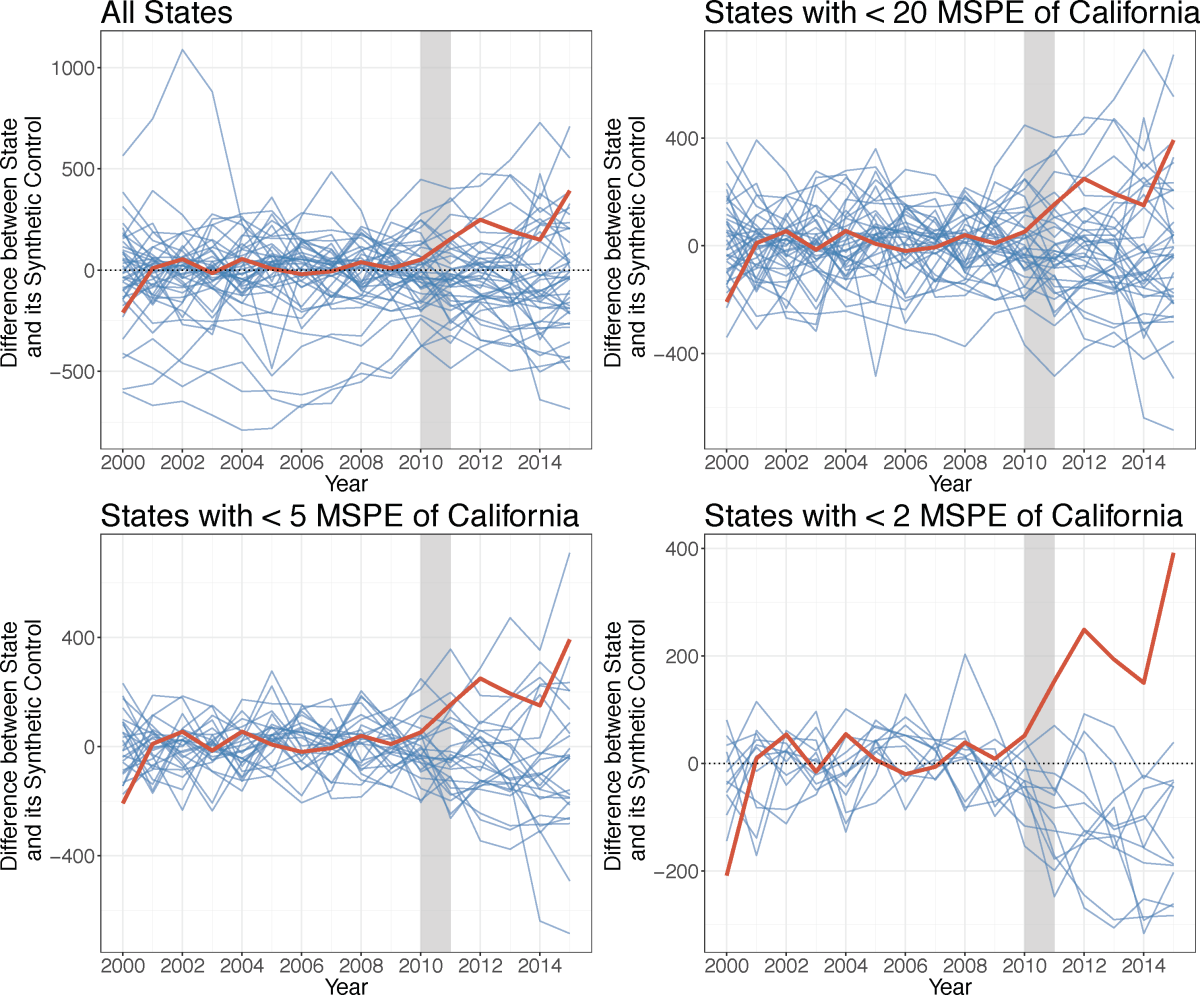
Differences between property crime rates for control states and California and their respective synthetic controls. Source: Property crime rates are from the FBI Uniform Crime Reports, 2000–2015. Note: The grey bar indicates the beginning of the drought.

Actual rates of property crime diverged from California’s synthetic control in 2011, increasing and peaking in 2012 and 2015, while remaining elevated over the control (Fig 2). The permutation test suggests this effect is significant compared to other states—although only 13 states had MSPE less than or equal to twice that of California, the effect observed in California was the greatest (Fig 3). The differences in property crime between California and its synthetic control from 2011–2015 indicate there was an average increase in property crime during the drought of approximately 9.65 percent.

**Fig 4:**
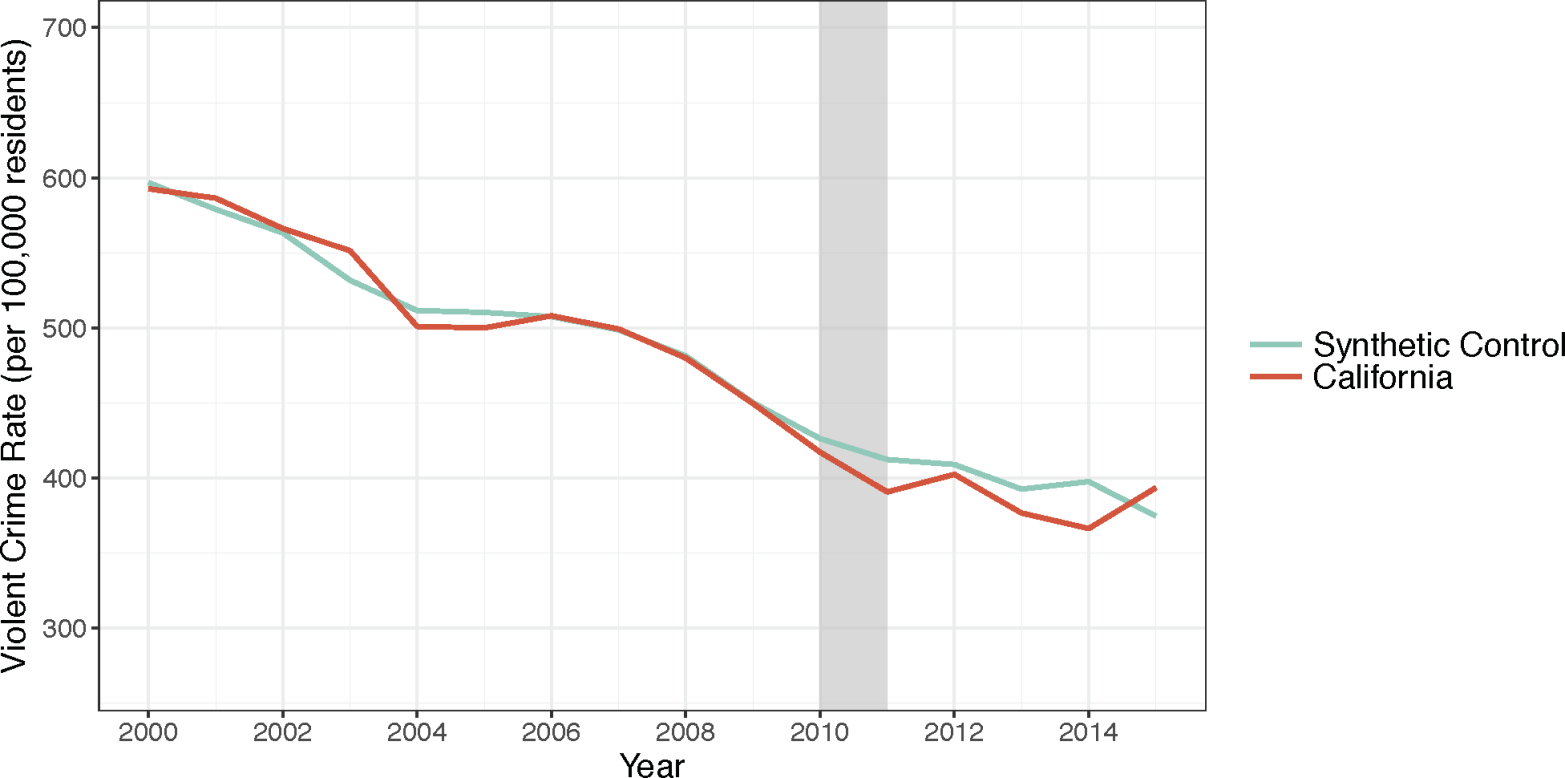
Violent crime in California and its synthetic control. Source: Violent crime rates are from the FBI Uniform Crime Reports from 2000–2015. Note: The grey bar indicates the beginning of the drought.

**Fig 5:**
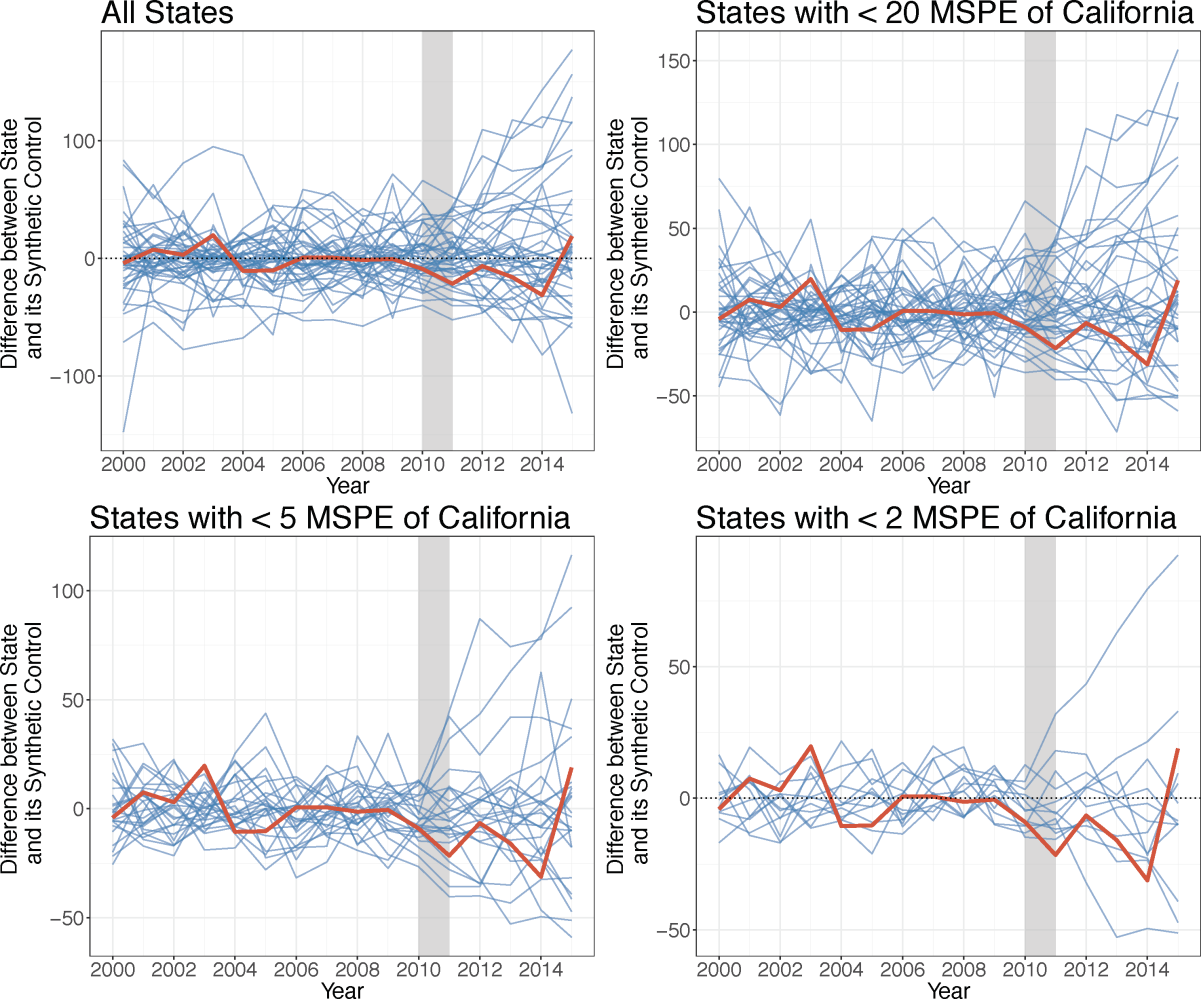
Differences between violent crime rates for control states and California and their respective synthetic controls. Source: Violent crime rates are from the FBI’s Uniform Crime Reports, 2000–2015. Note: The grey bar indicates the beginning of the drought.

Actual rates of violent crime in California declined from 2000–2010, and continued to decline during the period of the drought, similar to the estimated pattern of the synthetic control (Fig 4). The actual violent crime rate was slightly below the level predicted by the synthetic control. The differences between the observed and predicted values were similar to those seen in other states, even among states that had MSPE less than twice that of California (Fig 5). It does not appear that there was a significant effect of the drought on violent crime rates. Our sensitivity analysis including temperature as a covariate did not change either the property crime or violent crime results.

**Fig 6:**
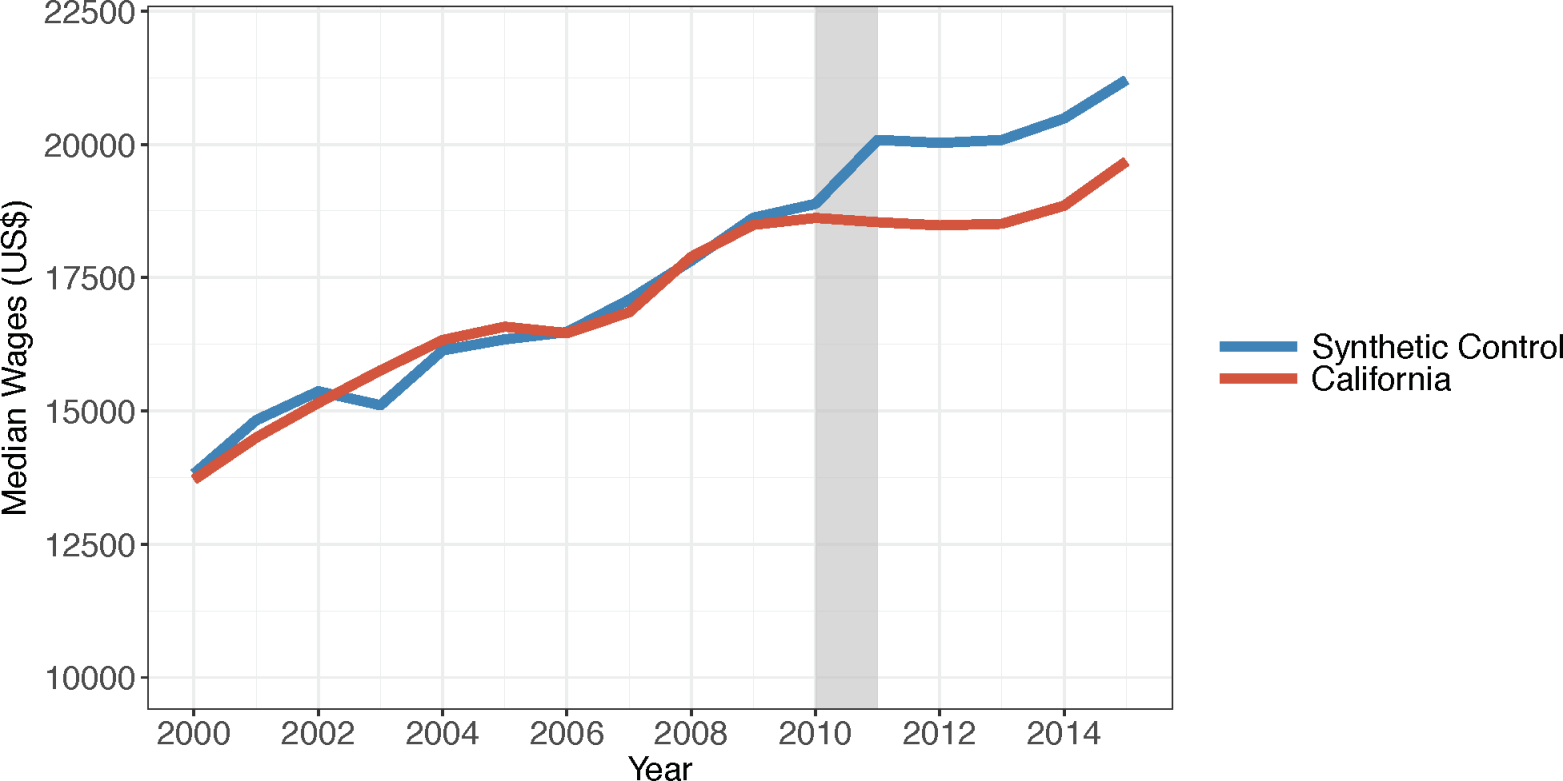
Median farmworker annual wages in California and its synthetic control, 2000–2015. Source: Median farmworker wages are from the Bureau of Labor Statistics Occupational Employment Statistics program. Note: The grey bar indicates the beginning of the drought.

**Fig 7:**
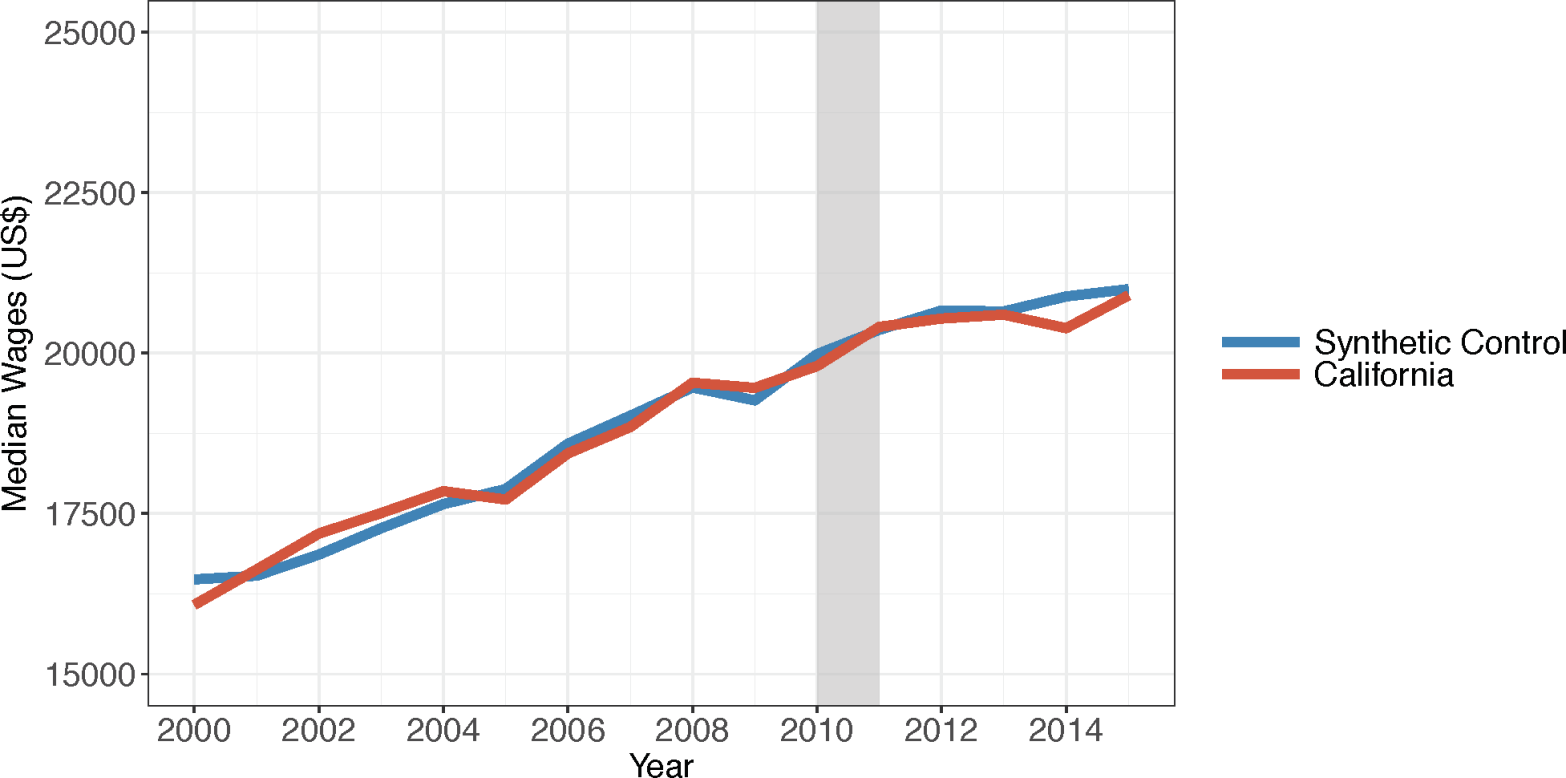
Median cashier annual wages in California and its synthetic control, 2000–2015. Source: Median cashier wages are from the Bureau of Labor Statistics Occupational Employment Statistics program. Note: The grey bar indicates the beginning of the drought.

To explore alternate explanations for the impact of drought on property crime, we used a negative control and what we called a positive control to assess the drought’s impact on median cashier and farmworker annual wages. The direction of the results suggests drought may have depressed farmworker wages (Fig 6), although the results are not very unusual according to the permutation test. Of the 15 states with MSPE less than twice California’s, 2 states (or 13%) had results more extreme than California’s (data not shown). There are no apparent differences between the median cashier wages in California and its synthetic control during the drought period (Fig 7). Of the 35 states with MSPE less than twice California’s, 8 (or 23%) had a result more extreme (data not shown). These results suggest that economic conditions not controlled in our covariates that were also unrelated to the drought were unlikely to have caused our results.

## Discussion

We found evidence that the drought in California was associated with an increase in property crime but was not associated with a change in violent crime relative to the synthetic control. An increase in property crime during the time of the drought is consistent with the routine activity theory of crime, suggesting increased opportunity for burglary, motor vehicle theft, or larceny theft to occur, potentially coupled with exacerbated economic and social discontent, shifting incentives for property crime and altering the rate of victimization.

We expected to see an increase in violent crime during the drought based on previous studies, but our results suggest that violent crime was not affected by the drought. It is possible violent crime is not as susceptible to weather changes, or that drought-related economic pressures were not widespread enough during 2011–2015 to increase violent behavior. There could also have been policies or violence prevention programs initiated during 2011–2015 that mitigated the effects of drought. It is also possible that competing influences of the drought on routine activities and weather-related behavior cancelled out any effect.

This analysis has several limitations. While the synthetic control method allowed us to capture the desired scope of impacts from the drought, it does not test how each mechanism—economic changes or alternations in routine activities—may have individually contributed to the crime rate. In addition, the exposure of interest was represented as a dichotomous indicator of drought, to encompass the complex social and economic consequences of the drought. Thus, there is likely to be some misclassification. Furthermore, because we used state-year as the unit of analysis, it is possible our analysis has overlooked possible differences in seasonal effects, or differential effects by rurality. We hope these factors are explored in future work. In addition, we attribute any differences between the observed and expected crime rates to drought, but it is possible another event not captured in our covariates co-occurred in 2011 and continued through 2015 that caused property crime to increase over that time. We used a negative control to address this concern, and found minimal evidence that the effect was due to economic changes unrelated to drought and not included in our covariate list. However, the possibility remains that some other event or societal changes were responsible for the increase in property crime. Such changes would need to have occurred statewide, or resulted in an extreme increase in crime in one locality to such an extent that it became identifiable in the state crime rate. The event would need to have occurred in California beginning in 2011 but not in states selected as part of California’s synthetic control. We believe such an event is unlikely.

The inferential techniques used to assess the significance of the estimates from the synthetic control method are exact, and thus do not have the asymptotic properties or interpretation of theory-based inference. However, with serially correlated data, exact inference is preferred, as theory-based inference is usually biased [63]. In addition, due to the case-comparison nature of the study, permutation-based inference is more appropriate than methods that rely on large samples [63]. Finally, other states experienced drought at the beginning of 2011, although none experienced the severe and persistent drought as California did. If states in the synthetic control also suffered drought-related crime effects during the 2011–2015 period, the estimates provided here can be considered conservative.

The results and inference from the synthetic control method assume that California’s drought did not affect violent or property crime rates in other states. We do not believe that California’s drought impacted crime rates in other states, but if it did, neighboring states would be the most likely to be impacted. As a sensitivity analysis, we excluded all states bordering California from the control pool, and results were similar to those presented above.

This study also benefits from several strengths. We used 10 years of pre-drought data to establish crime trends in California before the drought. Due to the dynamic nature of the drought’s impact on social and economic behaviors, we modeled drought as an environmental event with other states as controls. This allowed us to capture effects of the drought that may have been obscured by another analysis method. By choosing state-year as the unit of analysis, we were able to include many covariates, improving the quality of the estimates from the control group. This analysis was motivated by a discussion of the mechanisms theorized to link annual variations in climate to crime and discussed their relevance in the California context.

We utilized a synthetic control method that improved our control group selection, accounted for the autocorrelation of crime and drought, and estimated the impact of drought on crime each year from 2011–2015. A difference-in-differences approach is often used to compare outcomes between two groups before and after an exposure or intervention, but this approach is limited in several ways. Specifically, there are three key issues that motivated our use of an alternative, synthetic control method. First, a difference-in-differences approach could conflate differences in crime associated with drought with differences due to pre-drought characteristics associated with drought if the control group is not similar enough to California. The synthetic control method is guaranteed to be at least as similar to California as a simple weighted mean of control states or any one other control state [49]. Second, traditional difference-in-differences methods assume unobserved confounders are time constant, whereas the effects of observed and unobserved pre-drought confounders do not have to be time constant in the synthetic control method [64]. Third, traditional difference-in-difference methods provide asymptotic large-sample inference that is inappropriate given the comparative case study design, whereas the synthetic control method uses exact inference [63].

As communities in California and throughout the country plan for a future of climatological uncertainty, it is important to acknowledge the potential of climate and the weather to impact human behavior. Our results indicate that California’s drought was associated with an increase in property crime, but we did not find an effect of the drought on violent crime. As the drought continues, however, there are likely to be substantial economic impacts, especially for farmworkers and other vulnerable populations in California. These structural changes may exacerbate economic disempowerment or political dissent that incites violence. Such effects have been observed in other regions around the world, and it is unclear how resilient California will be to these challenges.

Future studies should explore the mechanisms that link climate and weather to crime by assessing changes in routine activities during drought and their relationship to crime victimization. Additionally, as the drought continues in California, it will be important to assess the extent to which unemployment, water prices, groundwater shortages, and other economic factors are affected, and to examine whether these factors are associated with changes in criminal behaviors. For now, social scientists and policy analysts engaged in forecasting the impact of climate change should consider the possible impact of altered climate conditions on crime, especially property crime.

## Supporting information

S1 File Dataset for drought and crime synthetic control analysis, 2000–2015. (CSV)
